# [Retracted] Dual inhibition of COX‑2/5‑LOX blocks colon cancer proliferation, migration and invasion *in vitro*

**DOI:** 10.3892/or.2024.8845

**Published:** 2024-11-18

**Authors:** Xiao-Hang Che, Chun-Lin Chen, Xiao-Lei Ye, Guo-Bin Weng, Xian-Zhi Guo, Wen-Ying Yu, Jin Tao, Yi-Chen Chen, Xiaodong Chen

Oncol Rep 35: 1680–1688, 2016; DOI: 10.3892/or.2015.4506

Following the publication of the above paper, it was drawn to the Editor's attention by a concerned reader that there appeared to be a number of anomalies in the presentation of data, including both tabulated data and data in figures: First, a discrepancy was noted between the numbers of males and females reported in the Materials and methods section on p. 1681 (47 females and 47 males) and yet, in Table I, showing clinicopathological characteristics of the patients, different numbers of male and female patients were reported. Secondly, data in Fig. 1 on p. 1683 had been misassembled: Experiments with COX-2 and 5-LOX are shown, but the panels for the ×40 and ×100 magnified images are presented the wrong way around. Thirdly, regarding the western blotting data, there were a couple of instances where it appeared that different exposures of gels had been undertaken in an attempt to show the same data for different experimental conditions. Fourthly, western blotting data featured internally in Fig. 4C on p. 1686 appeared to be strikingly similar, such that the same data may have been used to show the results for differently performed experiments. Fifthly, the data shown in the left-hand columns for the scratch-wound assay experiments in Figs. 2A and 5A were the same, even though different experiments were intended to have been portrayed in these figures. Finally, after having conducted an independent investigation of this paper in the Editorial Office, the presence of overlapping sections was also noted comparing the Transwell cell invasion assay data in Figs. 3 and 5.

Given the identification of these various issues in the paper, the Editor of *Oncology Reports* has decided that this article should be retracted from the Journal on account of the uncertainties, and an overall lack of confidence, in the presented data. The authors were asked for an explanation to account for these concerns, but the Editorial Office did not receive a reply. The Editor apologizes to the readership for any inconvenience caused.

